# The persuasive effects of social cues and source effects on misinformation susceptibility

**DOI:** 10.1038/s41598-024-54030-y

**Published:** 2024-02-20

**Authors:** Cecilie S. Traberg, Trisha Harjani, Jon Roozenbeek, Sander van der Linden

**Affiliations:** Department of Psychology, School of the Biological Sciences, University of Cambridge, Downing Street, Cambridge, CB2 3EB UK

**Keywords:** Human behaviour, Psychology

## Abstract

Although misinformation exposure takes place within a social context, significant conclusions have been drawn about misinformation susceptibility through studies that largely examine judgements in a social vacuum. Bridging the gap between social influence research and the cognitive science of misinformation, we examine the *mechanisms* through which social context impacts misinformation susceptibility across 5 experiments (*N* = 20,477). We find that social cues *only* impact individual judgements when they influence perceptions of wider social consensus, and that source similarity *only* biases news consumers when the source is high in credibility. Specifically, high and low engagement cues (‘likes’) reduced misinformation susceptibility relative to a control, and endorsement cues increased susceptibility, but discrediting cues had no impact. Furthermore, political ingroup sources increased susceptibility if the source was high in credibility, but political outgroup sources had no effect relative to a control. This work highlights the importance of studying cognitive processes within a social context, as judgements of (mis)information change when embedded in the social world. These findings further underscore the need for multifaceted interventions that take account of the social context in which false information is processed to effectively mitigate the impact of misinformation on the public.

## Introduction

Although social networking sites were initially created to facilitate interpersonal communication and social interactions between users, these platforms have also become one of the main facilitators of misinformation^1–4^. Unfortunately, as countless studies and reports have documented, misinformation can have dire consequences for the political and social wellbeing of societies around the globe^5–8^. While researchers studying misinformation have made insightful contributions to our understanding of who is most susceptible to misinformation^9–11^, seminal papers have made conclusions about what makes people susceptible by studying misinformation mostly in a social vacuum, when in reality, individuals often consume news in social settings. Social information represents a ubiquitous and virtually ineluctable part of the internet, with news consumers consistently being exposed to contextual cues accompanying news^12–15^, and most news consumers (including half of US adults)^16^ get at least some—if not an increasing share—of their news from social media^17–19^. As Jaeger and Burnett^20^ pointed out:*“Theoretical frameworks for understanding information within a social context are frustratingly rare”* (*p.* 4), and we posit that this remains the case today.

**Figure 1:**
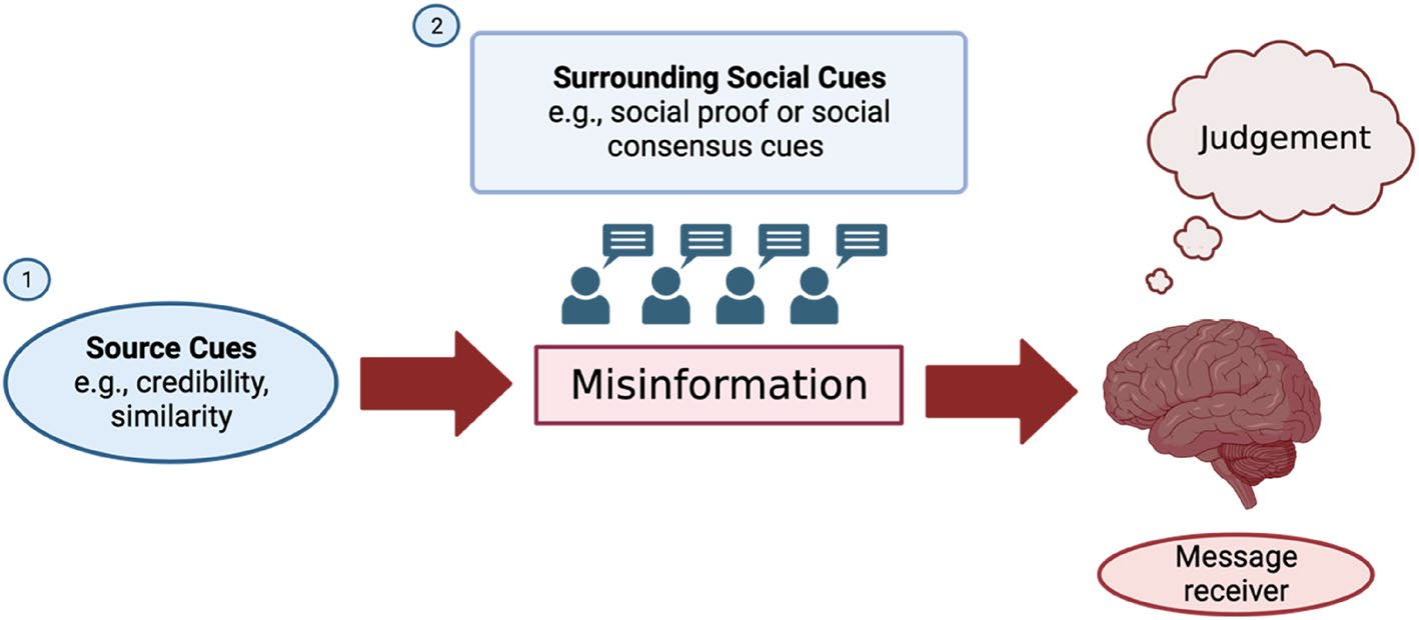
The social context of misinformation. (**1**) Misinformation content originates from a source for which source cues are present. (**2**) Surrounding social cues may be present in the form of social proof (e.g., ‘likes’ or comments) or social consensus cues (e.g., explicit judgements of others).

When consuming news, there are two main contextual elements individuals may be exposed to while they make judgements about headline veracity: Firstly, the source of the information, and secondly, the surrounding social cues (see Fig. 1). Although some studies have sought to examine whether both contextual cues impact misinformation susceptibility, results are highly mixed^12,21–24^. That is, in some circumstances, social cues (or ‘social proof’^25^) seem to influence individual susceptibility to misinformation, while at other times they do not. Under some circumstances, source effects have been found to play a role in misinformation susceptibility^24,26^, whereas in other contexts they have not^27^. More importantly however, to the best of our knowledge, no previous research has examined *how*, that is, through which underlying cognitive mechanisms social and source cues impact susceptibility. In this work we specifically ask: what are the cognitive processes through which individual misinformation susceptibility is impacted via social and source cues? Under which circumstances do social cues impact individual misinformation susceptibility? When do sources bias news consumer judgements of misinformation? Below, we expand on previous research and propose the mechanisms through which this influence may occur.

### Social cues and misinformation susceptibility

As social media sites represent ecosystems of social information where users can explicitly indicate agreement with or interest in information, express their opinions regarding news, and share information with other users^20^, some of these social cues may signal to other users that interest or support for the given information is high. Previous research has found that social or group consensus, defined as *“the proportion of a population who support a particular attitudinal position”*^28^ (*p. 1*) can have a powerful influence on individual attitudes, and has been named one of the most important factors in determining whether people conform to others^28–30^. For example, social consensus has been found to influence attitudes towards the legalisation of voluntary euthanasia^28^, and to mediate attitudes towards climate change^31^. Furthermore, estimates of social consensus (which we in this paper refer to as ‘perceived consensus’) predict scientific beliefs, and the presentation of public consensus information influences scientific beliefs^32^. Previous research has also distinguished between *implicit* and *explicit* consensus, referring to implicit consensus as beliefs about what others would do if they were present, and explicit consensus as the actual behaviour of others (Kassin, 1979, *p 1967*), showing that both these two forms of consensus influence individual level beliefs^33,34^.

In the context of misinformation, social engagement cues on social networking sites such as ‘likes’ have in some research been found to increase the perceived credibility of misinformation^13,23^, whereas other research found no effect of such cues on perceived message credibility^21^. Moreover, previous credibility ratings have been found to have no effect on perceived news credibility, but negatively valenced comments calling out misinformation made it less believable than when no comments were present^35^.

As such, while previous research has sought to uncover whether or not certain social cues influence misinformation susceptibility, results are mixed. Moreover, it is currently unknown through which mechanism this influence occurs. Why do social cues influence perceived reliability of misinformation in some instances but not others? Some researchers have speculated that social cues such as ‘likes’ may lack a negative social interpretation^22^; and therefore do not serve as any meaningful representation of perceived social consensus. That is, because ‘likes’ on platforms such as *X* (previously *Twitter*) and *Facebook* are not contrasted with a ‘dislike’ button, news consumers may not see these engagement cues as representing a direction-related sentiment (e.g., belief in or agreement with a certain social media post).

In this paper we investigate the underlying mechanism of perceived consensus, testing whether social cues impact perceptions of wider societal consensus and subsequent individual-level judgements. We firstly ask whether social engagement cues such as ‘likes’ impact individual judgements, but more importantly, we ask whether these cues are seen as representative of larger societal consensus. We further investigate whether individuals are influenced by both exposure to implicit group consensus information (e.g., comments which simply *imply* endorsement of a headline’s veracity, such as through reacting with concern to a news headline), and explicit group consensus, that is, information which explicitly details the judgements of a group^29^. Finally, we examine whether these implicit and explicit ‘local’ group cues impact perceptions of wider public consensus, and whether public consensus beliefs influence individual-level judgements.

### Source cues and misinformation susceptibility

A second way in which consuming news in a social context, such as on social media, may influence the individual is through source cues^36–38^. Early work on social influence has demonstrated that politically similar sources are more influential than dissimilar sources when it comes to persuasive messages^39^, and we are more likely to be persuaded by^40^ and accept advice^41^ from similar or like-minded others. This similarity can be inferred via group identity cues such as political ideology, or cues that indicate other forms of attitudinal similarity^42,43^.

There is already some evidence that source cues on social media influence how individuals judge information veracity. In one study, individuals judged misinformation as more likely to be true if it was attributed to sources that had previously published attitudinally congruent news^44^. Other research has found that Republican supporters of former US President Donald Trump were more likely to believe misinformation attributed to the previous President compared to misinformation presented without a source^45^. Another study revealed that participants were more likely trust information shared by elite individual sources they had previously deemed trustworthy compared to those they had deemed untrustworthy. Interestingly, informing them whether the article originated from a real or fake news outlet later had no impact on their trust ratings of the information^46^, a finding echoed by other research^47^. Indeed, although some research has found that source cues influence perceived veracity of (mis)information, other research has highlighted that simply emphasising news sources does not reduce misinformation susceptibility^27^. Furthermore, in a study manipulating both the political slant of true news content and source, the political slant of sources was not found to play a role in whether or not partisans judged headlines as true^48^. However, when investigating the impact of sources on susceptibility to non-partisan misinformation, recent research found that individuals were more susceptible to misinformation from US news outlets that shared their political identity, an effect driven by perceived source credibility^26^. However, being more likely to believe misinformation from real news sources that share one’s political identity may be based on past experience with those sources, as some news outlets have been shown to publish more misleading news than others^49,50^. This begs the question of whether people fall for misinformation attributed to similar sources *simply* because they prefer to get information from similar sources *even* if they are not credible. In this paper we examine whether source similarity effects exist even if people are presented with information indicating that the similar source lacks credibility.

### The present studies

Across 5 experiments, we set out to examine to what extent and how the social context, including social cues and source cues, impacts misinformation susceptibility. In the first set of studies (studies 1a, 1b & 2) we investigated whether and how social cues and perceived consensus impacts individual judgements of misinformation. Each of these studies aimed to test whether social cues increase individual susceptibility to misinformation (H1), and 1b and 2 tested whether such cues imply a wider social consensus in the belief that misinformation is reliable (H2). We further tested whether perceived consensus in the reliability of misinformation predicts (H3) and mediates (H4) the effect of social cues on the perceived reliability of misinformation. We pre-registered the hypotheses in study 2 (https://osf.io/4yn7z?view_only=00dca2d0bcfc42bc9393c2f6c7090d41).

The second set of studies (studies 3–4) set out to examine the causal and interactive impacts of source similarity and credibility, testing the main hypothesis that source similarity increases misinformation susceptibility (H5) and that both source similarity (H6) and credibility factors (H7) independently increase misinformation susceptibility, even for fictitious news sources. Of these studies, the second (study 4) was pre-registered (https://osf.io/wk9ya/?view_only=62174d2be7d64adaa17814386bbc5e1c). To broaden the generalisability of results, we use a wider set of stimuli that is broad in content, and in contrast to previous research, we use a deception-based definition of misinformation. That is, we define misinformation by whether or not the content makes use of deception techniques, rather than defining it as content that is blatantly false or from bogus sources. Although we tested hypotheses across multiple studies, we did not pool the data for any analyses. All data is available at on OSF^34^.

## Results

### Studies 1a, 1b & 2

The purpose of studies 1a, 1b & 2 was to test the impact of social cues on the perceived reliability of misinformation. Specifically, we investigate whether various social cues signal the beliefs of others (perceived consensus), and assess how these cues impact individual judgements. The structure of the three studies was similar. Participants were exposed to a series of false headlines and asked to make judgements regarding these. A full overview of headlines used in each study can be found in Supplementary Information Tables A–E. A description of how headlines were selected and/or developed is found in the Methods section.

In each of the studies we experimentally manipulated the social cues surrounding the headlines. Study 1a tested whether the social cues of ‘likes’, ‘retweets’ & ‘comment’ numbers impact misinformation susceptibility. Data was collected using an open access internet platform as part of a larger intervention experiment (*N* = 7788, 58% between 18 and 29, 39% female, 85% completed at least some college, 48% left-leaning), and participants were assigned to one of three conditions: A ‘high’ condition, where the headlines appeared to have a high number of likes, retweets and comments, a ‘low’ condition, where these numbers were low, or a control with these cues cropped out. Study 1b tested whether the same social cues impacted perceptions of wider social consensus (*N* = 628, *M*_age_ = 37, 68% female, 50% university educated, 52% left-leaning).

**Figure 2:**
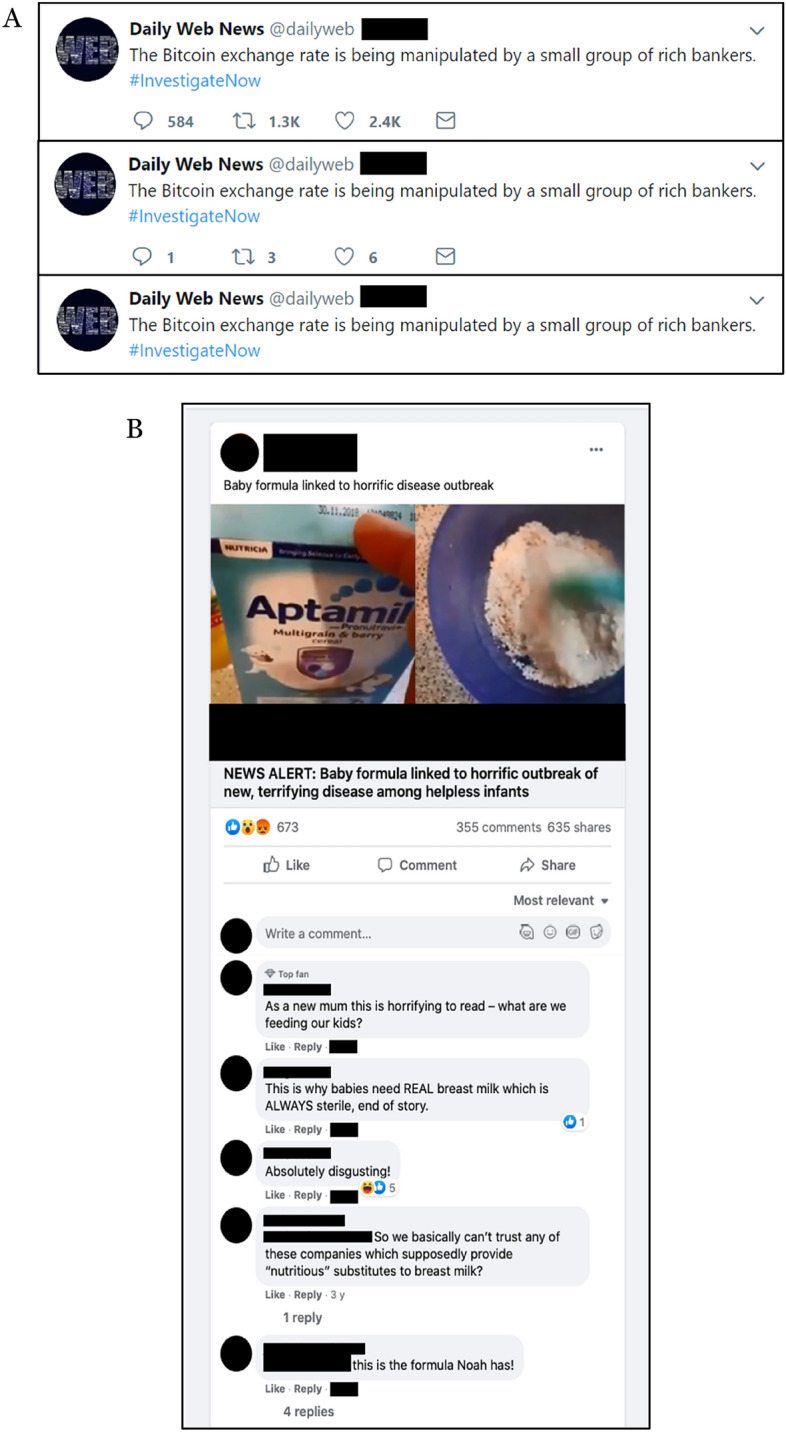
Example of headlines used in studies 1a and 2. (**A**) Study 1a example of item across social cue conditions: High (top), low (middle), control (bottom). (**B**) Examples of item in comment endorsement condition in study 2. *Note*: We use the Aptamil image under fair use that allows the use of copyrighted material under certain circumstances, such as for criticism, commentary, news reporting, teaching, scholarship, and research.

In study 2 (*N* = 730, *M*_age_ = 27, 46% female, 53% university educated, 64% left-leaning), data was collected via *Prolific *and tested the impact of implicit and explicit social cues on individual misinformation susceptibility and perceived consensus. We manipulated social cues across 5 conditions: *Implicit* consensus, manipulated through comments endorsing (i.e., indicating belief in) misinformation vs discrediting misinformation, *explicit* consensus manipulated through direct percentages of previous study participants endorsing (i.e., indicating belief in) vs discrediting misinformation, and a control condition. More specifically, implicit ‘endorsement’ was operationalised through comments: Participants were exposed to comments underneath each headline that implicitly indicated the commenters believed the headline to be true. That is, the comments did not directly say things like “*This is reliable!*” (as has been the case in previous research)^35^, but instead used other means to suggest they may find it reliable, e.g., through shock: “*As a new mum this is horrifying to read- what are we feeding our kids*?” or drawing conclusions based on the statement in the headline, e.g.: “*This is why babies need real breast milk which is always sterile, end of story*”. Similarly, implicit discrediting was operationalised through comments that implicitly suggested the commenters found the headline unreliable, e.g.: “*Terrifying? I heard it’s just a cough*”. See Fig. 2 for an example of materials in studies 1a and 2 (implicit endorsement condition).

#### H1: Level of engagement and discrediting cues had no effect, but endorsement cues increased misinformation susceptibility

To test whether social cues online impact perceived reliability of misinformation (H1), we collected data on the perceived reliability of misinformation across the different social cue conditions and conducted one-way ANOVAs. Average perceived reliability of all misinformation headlines was calculated separately for each study.

In study 1a we find a significant effect by group (*F*(2,7785) = 42.64, *η*^2^ = 0.01, *p* < 0.001), but contrary to our hypothesis, post-hoc tests revealed that perceived reliability of misinformation was lower in both the high ‘likes’ condition (*M* = 2.68, *SD* = 1.36, *p* < 0.001, *d* = − 0.28) and in the low ‘likes’ condition (*M* = 2.76, *SD* = 1.34,* p* < 0.0001, *d* = − 0.22) compared to the the control condition (*M* = 3.07, *SD* = 1.64), but there was no significant difference between the high and low conditions (*p* = 0.20, *d* = − 0.05).

In study 1b the social cue manipulation was not significant in the main model (*F*(2,625) = 2.58, *η*^2^ = 0.01, *p* = 0.08), but the pattern was similar to study 1a, with reliability being lower in both the high likes (*M* = 2.64, *SD* = 0.77, *p* = 0.15, *d* = − 0.18) and low likes (*M* = 2.62, *SD* = 0.83, *p* = 0.10, *d* = − 0.20) than in the control condition (*M* = 2.78, *SD* = 0.78).

In study 2, as pre-registered, analyses were run with both the full sample and when excluding participants who did not pass the attention check. Analyses run with the full sample (*F*(4,725) = 6.35, *p* < 0.0001) show a significant main effect of social cues on perceived reliability. Planned contrasts showed that participants judged misleading headlines as more reliable when previous participant judgements endorsed the misinformation (*M* = 2.83, *SD* = 1.23) compared to when they discredited it (*M* = 2.23, *SD* = 1.01, *p* < 0.001, *d* = 0.53). Participants also judged misleading headlines as more reliable when comments underneath endorsed the misinformation (*M* = 2.48, *SD* = 1.13) compared to when they discredited it (*M* = 2.30, *SD* = 1.10). This contrast was not significant in analyses run with the full sample (*p* = 0.18, *d* = 0.16), but was significant when excluding those who did not pass the attention check (*M*_*e*ndorse_ = 2.60, *SD*_endorse_ = 1.22, *M*_discredit_ = 2.23, *SD*_discredit_ = 0.99, *p* = 0.01, *d* = 0.34).

**Figure 3:**
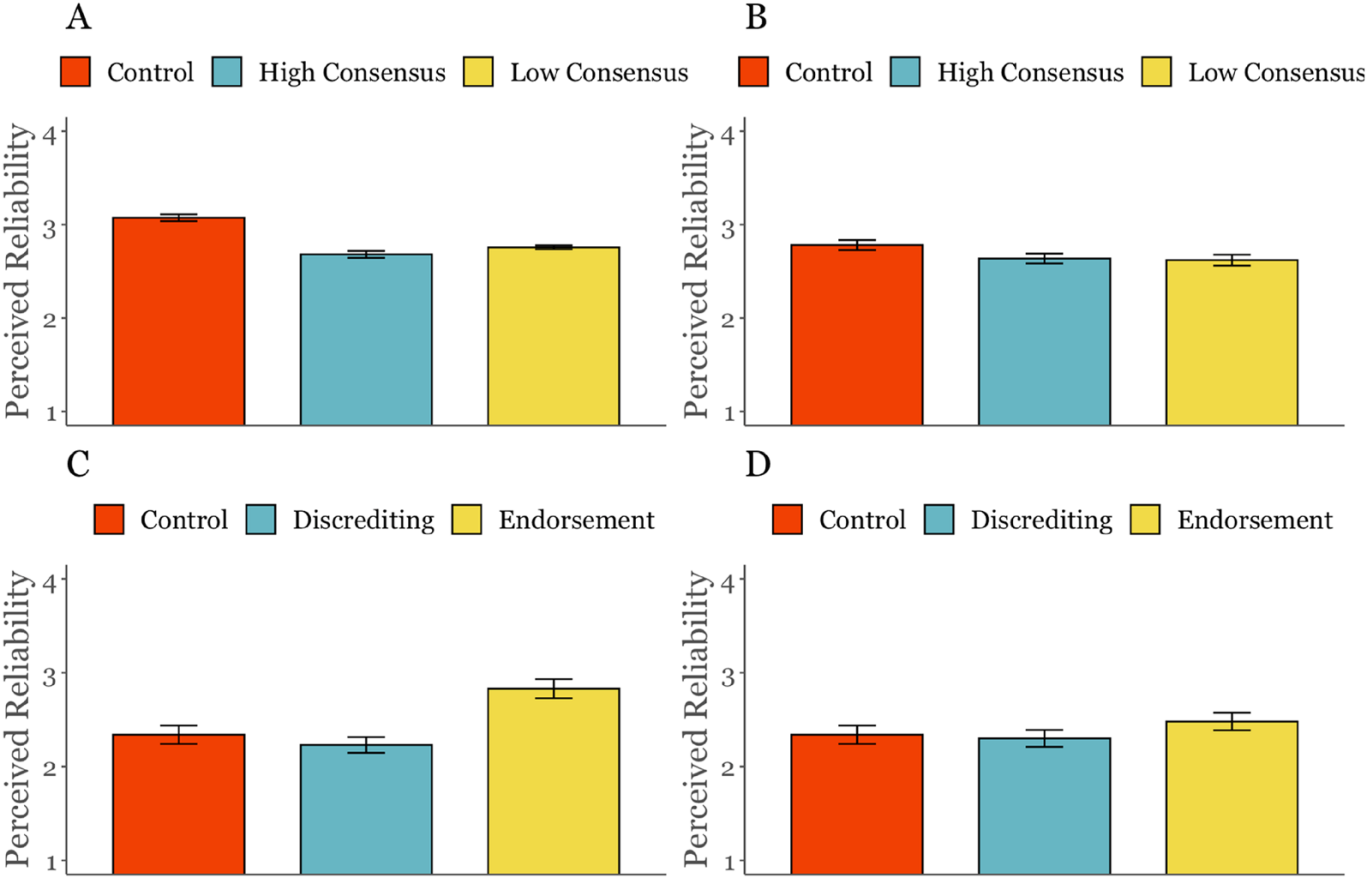
Studies 1a, 1b & 2: Mean perceived reliability averaged across misinformation headlines by study. (**A**) Likes, retweet & comment numbers in study 1a. (**B**) Likes, retweet & comment numbers in study 1b. (**C**) Previous participant judgements in study 2. (**D**) Comments in study 2. Error bars show standard error of the mean.

Further post-hoc tests revealed that there were no significant differences between both discrediting and the control conditions (*p’*s > 0.05), no significant differences between the comment endorsement and control (*p* = 0.81), but a significant difference between the previous participant endorsement (*M* = 2.83, *SD* = 1.23) and control condition (*M* = 2.34, *SD* = 1.20, *p* < 0.01, *d* = 0.43). After exclusions, there was however, a significant difference between the comment endorsement and control (*p* = 0.03, *d* = 0.43). Figure 3 shows the influence of social cue conditions across the three studies. A full list of results for analyses run after excluding participants who failed the attention check can be found in Supplementary Table F.

Across studies 1a, 1b and 2, we therefore only find partial support for H1. Level of engagement cues (e.g., high vs low ‘likes’) and discrediting cues had no effect on perceived reliability of misinformation, whereas endorsement cues increased belief.

#### H2: Endorsement and discrediting cues imply wider social consensus in the belief that misinformation is reliable

To test whether social cues imply a wider social consensus in the belief that misinformation is reliable (H2), we collected data on perceived consensus (study 1b & 2) across the different social cue conditions and conducted one-way ANOVAs. Average perceived consensus for all misinformation headlines was calculated separately for each study. Results revealed that in study 1b, likes, retweet and comment numbers had no effect on perceived consensus (*F*(2,624) = 1.01, *p* = 0.37). In study 2, however, social cues had a significant effect (*F*(4,725) = 51.84, *p* < 0.001). As specified in the pre-registration, we ran two contrasts. We first compared the comment endorsement and discrediting conditions, where perceived consensus judgements were significantly higher in the endorsement (*M* = 54.82, *SD* = 19.14) compared to the discrediting condition (*M* = 44.77, *SD* = 19.19, *p* < 0.001, *d* = 0.52). We subsequently compared the previous participant endorsement and discrediting conditions, where perceived consensus was also significantly higher in the endorsement condition (*M* = 61.22, *SD* = 16.93) compared to the discrediting condition (*M* = 32.38, *SD* = 14.77, *p* < 0.001, *d* = 1.81). Additional exploratory post-hoc tests show that both previous participant discrediting (*M*_diff_ = − 17.04, *p* < 0.001, *d* = 0.93) and endorsement (*M*_diff_ = 11.80, *p* < 0.001, *d* = 0.65) was significantly different to the control (*M* = 49.42, *SD* = 20.60), but there were no differences between the comment conditions and the control (*p*’s > 0.05). However, when excluding participants who failed the attention check, there was a significant difference between the comment endorsement condition and the control (*M*_diff_ = 8.56, *p* = 0.01, *d* = 0.33).

These results show partial support for H2. Here, engagement cues (e.g., ‘likes’) had no effect on perceived consensus, whereas both comments and previous participant judgements influenced perceived consensus.

#### H3: Perceived consensus in the reliability of misinformation predicts individual perceived reliability of misinformation

**Figure 4:**
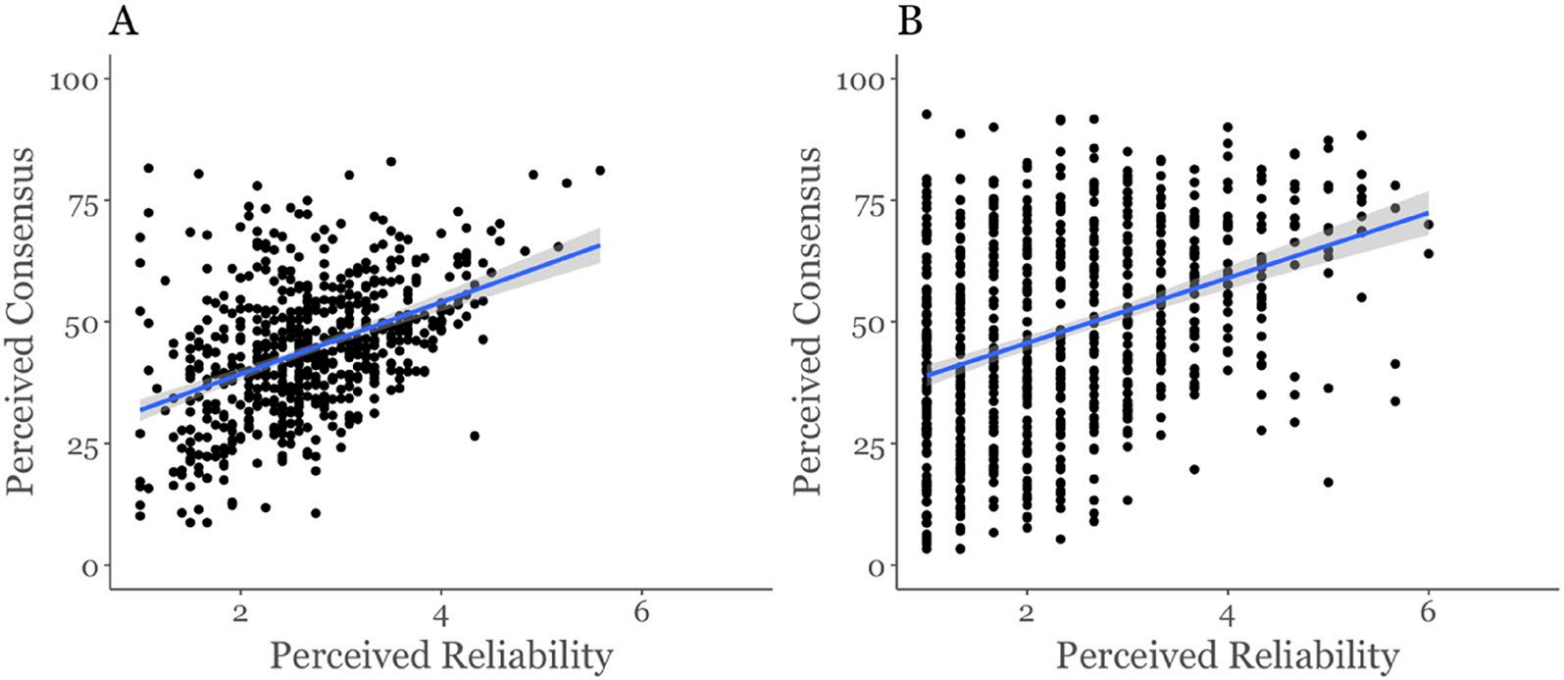
Mean perceived reliability by mean perceived consensus. (**A**) Perceived social media consensus in study 1b. (**B**) Perceived public consensus in study 2.

To test whether perceived consensus predicts perceived reliability of misinformation (H3) we ran two linear regression analyses with study 1b and study 2 data, using perceived reliability of misinformation as the DV and perceived consensus as the IV. Both studies find support for this hypothesis. In study 1b the regression was significant (Adjusted *R*^2^ = 0.19, *F*(1,625) = 143.8, *p* < 0.001), with perceived consensus predicting perceived reliability (*b* = 0.03, *p* < 0.001), and in study 2 the regression was also significant (Adjusted *R*^2^ = 0.14, *F*(1,728) = 118.2, *p* < 0.001), with perceived consensus predicting perceived reliability (*b* = 0.02, *p* < 0.001). Results for H3 are illustrated in Fig. 4. This suggests that when individuals perceive a majority of others to believe misinformation headlines, they too judge them to be reliable. Our results therefore support H3.

#### H4: Perceived consensus in the reliability of misinformation mediates the effect of social cues on perceived reliability of misinformation

To test H4 and investigate whether perceived consensus mediates the effects of social cues on perceived reliability of misinformation, we relied on study 2, as the social cue condition did not significantly predict perceived consensus nor perceived reliability of misinformation in study 1b.

To test perceived consensus as a mediator of the effect of explicit social cues on perceived reliability of false headlines, a mediation analysis was run with 10,000 bootstrapped confidence intervals in JASP using the SEM module. Here, the direct effect of social cue condition on perceived reliability was not significant with unstandardised regression coefficient *b* = − 0.03, *p* = 0.85, 95% CI [− 0.34, 0.27]. However, the indirect effect of social cue condition via perceived public consensus was significant, as the unstandardised regression coefficient for the mean bootstrapped indirect effect was *b* = 0.63, *p* < 0.001, 95% CI [0.43, 0.86]. The unstandardised regression coefficient for the total effect of social cue condition on perceived reliability was *b* = 0.60, *p* < 0.001, 95% CI [0.33, 0.85]. The effect of previous participant judgement social conditions on perceived reliability was thus fully mediated via perceived consensus.

To test perceived consensus as a mediator of the effect of implicit social cues on perceived reliability of false headlines, a mediation analysis was run with 10,000 bootstrapped confidence intervals in JASP using the SEM module. The mediation analysis revealed that the direct effect of implicit social cues on perceived reliability of misinformation was not statistically significant (*b* = − 0.03, *p* = 0.80, 95% CI [− 0.29, 0.23], but the indirect effect of the mediator of perceived consensus was (*b* = 0.21, *p* < 0.001, 95% CI [0.12, 0.34]). This suggests that the observed relationship between implicit social cues and perceived reliability was fully accounted for by the mediator. The total effect however, was not significant (*b* = 0.18, *p* = 0.16, 95% CI[− 0.07, 0.44]) suggesting a suppressed mediation. In suppressed mediation, the direct impact of social cues on perceived reliability is counteracted or suppressed by the mediating variable, perceived consensus. The non-significant total effect thereby stems from the opposing signs of the direct (negative) and indirect (positive) paths, leading to their effects offsetting each other. Importantly, this doesn’t discount the observed significant mediation^51^, emphasising the need for a more nuanced consideration when understanding the impact of social cues on perceived reliability of misinformation. Analyses conducted with exclusions applied showed the same pattern of results, albeit with a significant total effect (*p* = 0.02). As such, results fully support H4.

Together the mediation models suggest that both implicit cues, such as comments, and explicit cues, in the form of previous participant judgements indicating the beliefs of a local group, influence perceptions of what the public believes, and this perception influences the perceived reliability of misinformation. To ensure that the full mediation findings were not statistical artifacts and results of strong correlations between the mediator and IV or DV, we ran correlation analyses, which are reported in Supplementary Information Table I.

#### The impact of social cues on perceived reliability of factual headlines

Following our main hypotheses, we further analysed the impact of social cues on perceived reliability of factual headlines. Results show that social cues played a role to some extent. Engagement cues (‘likes’) only played a role in perceived reliability of factual headlines in study 1a (*F*(2,7668) = 13.89, *p* < 0.001, *η*^*2*^ = 0.004), and not 1b (*F*(2,625) = 0.35, *p* = 0.70), but again in study 1a, there was no difference between high and low numbers of likes (*p* = 0.87), only between high numbers and the control (*M*_diff_ = − 0.27, *SE* = 0.06, *p* < 0.001, *d* = − 0.15) and low numbers and the control (*M*_diff_ = − 0.24, *SE* = 0.05, *p* < 0.001, *d* = − 0.13). In study 2, the social cues model was significant (F(4,725) = 11.01, *p* < 0.001, *η*^2^ = 0.06) with significant differences between the implicit discrediting and endorsement (*M*_diff_ = − 0.98, *SE* = 0.19, *p* < 0.001, *d* = − 0.62) and between the control and implicit discrediting (*M*_diff_ = 0.85, *SE* = 0.18, *p* < 0.001, *d* = 0.54). Interestingly, the contrast between explicit endorsement and explicit discrediting was not significant (*p* = 0.08). Looking at perceived consensus in factual headlines in study 2, a similar pattern to that of misinformation emerged. The main model was significant (*F*(4,725) = 17.3, *p* < 0.001, *η*^*2*^ = 0.09), with a significant difference observed between the control and the implicit discrediting conditions (*M*_diff_ = 13.23, *SE* = 2.22, *p* < 0.001, *d* = 0.69), between explicit discrediting and endorsement (*M*_diff_ = − 9.13, *SE* = 2.24, *p* < 0.001, *d* = − 0.48), and between implicit discrediting and endorsement (*M*_diff_ = − 13.23, *SE* = 2.23, *p* < 0.001, *d* = − 0.69).

### Studies 3–4

Studies 3 and 4 investigated whether source similarity and source credibility cues causally influence individual judgements of misinformation. Study 3 (*N* = 10,541, 60% between 18 and 29, 44% female, 49% higher educated, 52% liberal) data was collected as part of a larger intervention experiment on source cues, and manipulated the source of the misinformation headlines, such that it either contained sources (real liberal or conservative US news outlets) or a control condition which did not include any source. Following data collection, sources were coded as either being similar or dissimilar to the participants’ indicated political orientation to allow for factorial analyses. Moderates (*N* = 2417) were therefore excluded from these analyses.

**Figure 5:**
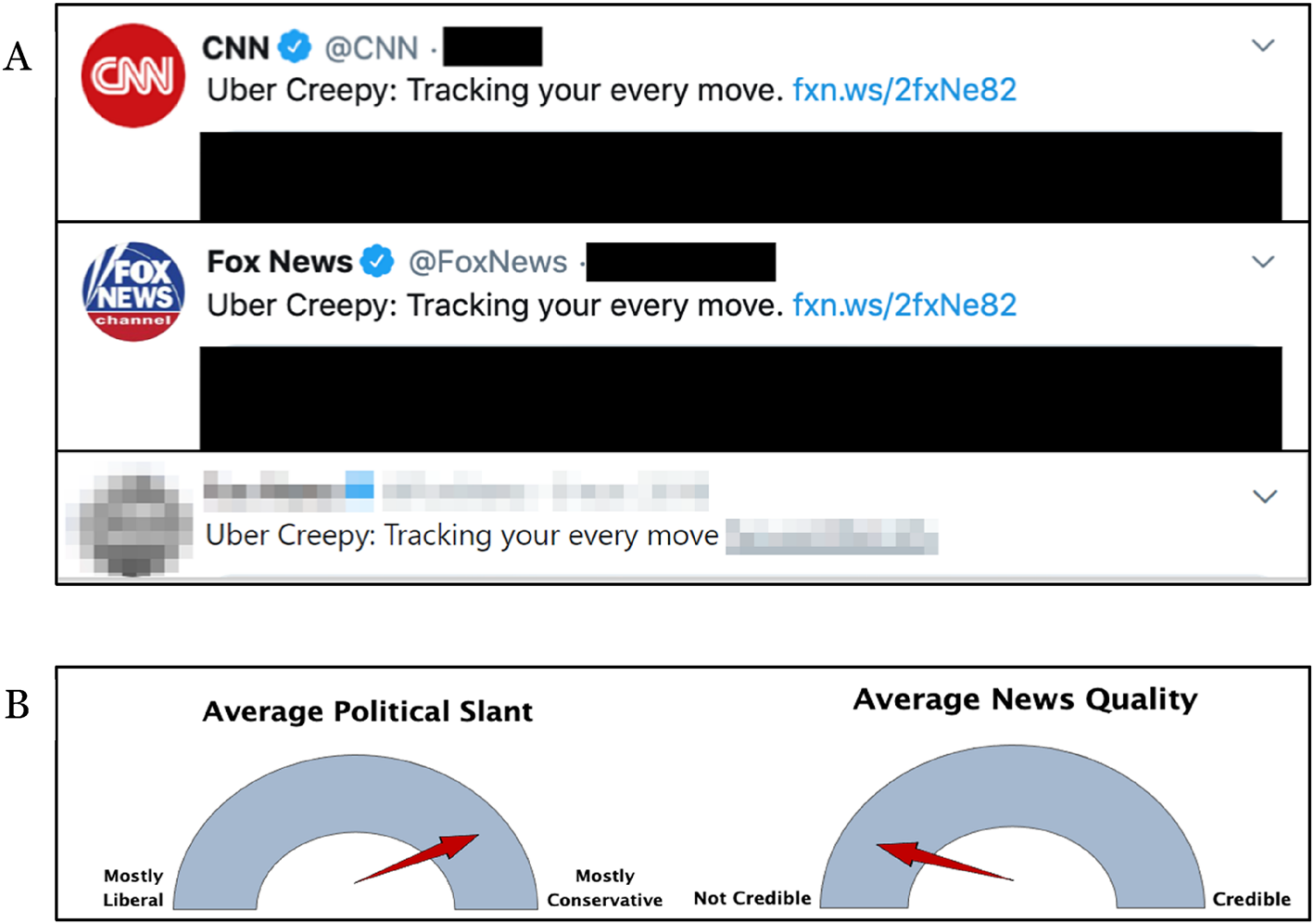
Example of source manipulations in studies 3 & 4. (**A**) News headline example in left-wing condition (top), right-wing condition (middle) & control (bottom) in study 3. (**B**) Example of source manipulation metres (conservative, low credibility condition) in study 4.

In study 4, we recruited *N* = 790 US participants who either identified as liberal or conservative (*M*_age_ = 35, 65% female, 62% university educated, 48% left-leaning). Participants made judgements about the reliability of news headlines following exposure to a vignette description and a graphical rating of the source, which was either described as liberally or conservatively slanted (subsequently coded as similar/dissimilar), and high vs low on credibility (2 × 2 design) (see Fig. 5).

#### H5: The presence of news sources influences the perceived reliability of misinformation

In study 3 we assessed the general influence of the inclusion of sources on misinformation headlines (H5). An independent samples t-test showed that participants who viewed false headlines without sources rated the reliability of these as significantly lower (*M* = 3.35, *SD* = 1.65) than those who viewed the headlines with sources (*M* = 3.57, *SD* = 1.39, *t*(10,539) = − 6.71, *p* < 0.001, *d* = − 0.15). As such, we find support for H5.

#### H6: Source similarity increases the perceived reliability of misinformation

As studies 3 and 4 manipulated source similarity, we examined H6 across both studies. In study 3, which employed a between subject’s design, participants viewed misinformation headlines from either liberally slanted real news sources, conservatively slanted real news sources or a control condition with no source. To investigate the effects of source similarity, we created a variable to code for whether the source matched participants’ political ideology. For this analysis, it was therefore necessary to exclude participants who identified as moderates.

**Figure 6:**
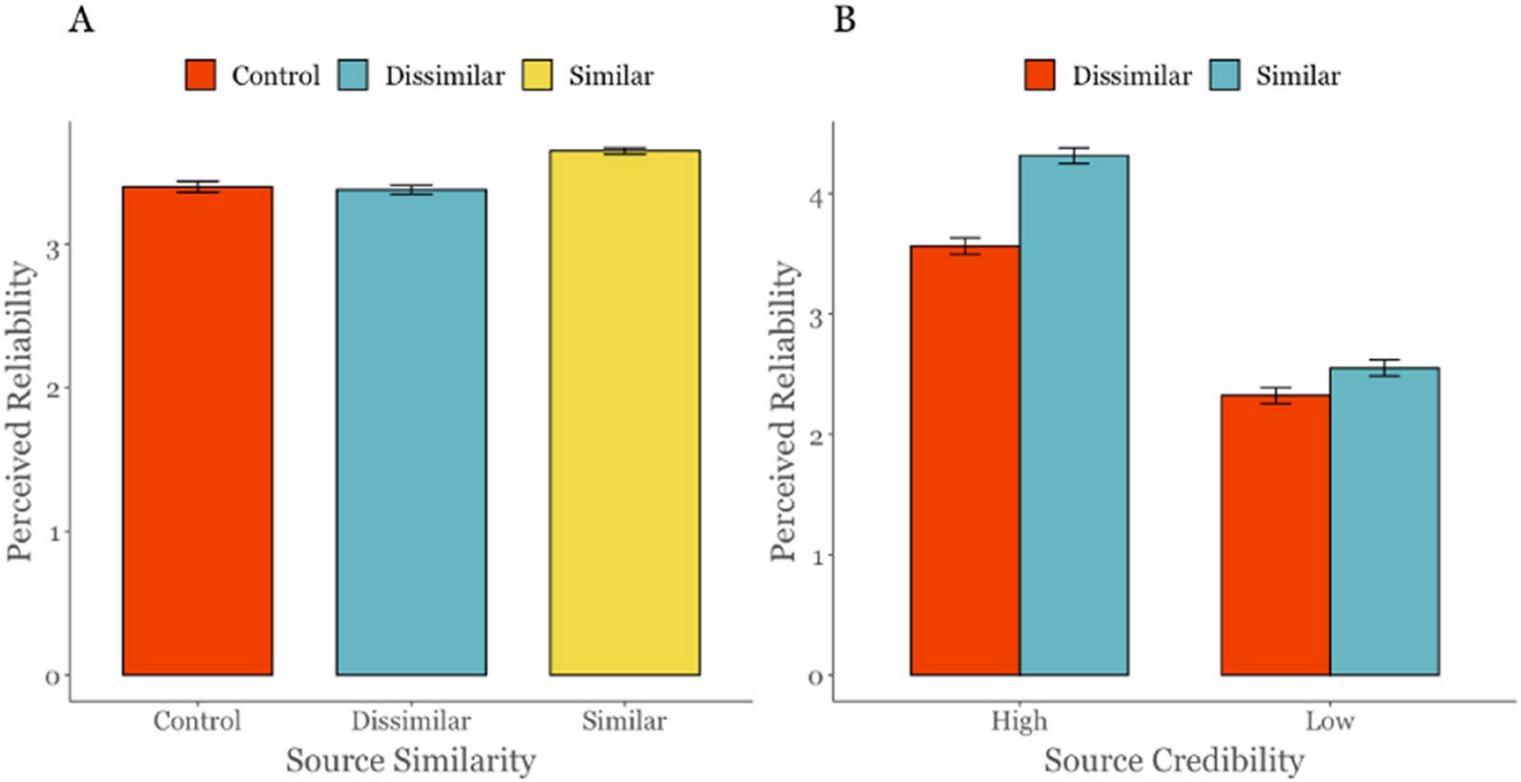
Source credibility and similarity effects on perceived reliability. (**A**) Perceived reliability of false headlines from real sources in study 3. (**B**) Perceived reliability of false headlines from fictitious news sources study 4. Error bars show standard error of the mean.

In study 3, a one-way ANOVA was run with similarity as the IV and perceived reliability of false headlines as the DV, showing a statistically significant effect of source similarity on perceived reliability,* F*(2,7695) = 31.31, *η*^2^ = 0.01, *p* < 0.001. Tukey’s post-hoc tests revealed significant differences between when the source was similar (*M* = 3.65, *SD* = 1.30) compared to when it was dissimilar (*M* = 3.38, *SD* = 1.48, *p* < 0.001, *d* = 0.19), and between when the source was similar and the control condition (*M* = 3.40, *SD* = 1.71, *p* < 0.001, *d* = 0.17), but no significant difference between when the source was dissimilar and the control condition (*p* = 0.83). Results of this analysis are visualized in Fig. 6.

To test H6 in study 4, a* t*-test was run comparing judgements of false headlines between the two source similarity conditions (similar vs dissimilar). As hypothesised, the analysis showed that participants rated misinformation from similar sources as being significantly more reliable (*M* = 3.46, *SD* = 1.28) compared to misinformation from dissimilar sources *(M* = 2.93, *SD* = 1.13, *t*(788) = 6.18, *p* < 0.001, *d* = 0.44).

H6 was thereby fully supported, as participants rated misinformation from politically similar sources as significantly more reliable than misinformation from politically dissimilar sources in both studies 3 and 4.

#### H7: Source credibility increases the perceived reliability of misinformation

To test H7 in study 4, a *t*-test was run comparing judgements of false headlines between the two source credibility conditions (high credibility vs low credibility). As expected, the analysis showed that participants rated misinformation from fictitious credible sources as being significantly more reliable (*M* = 3.94, *SD* = 1.10) compared to misinformation from fictitious sources lacking credibility *(M* = 2.43*, SD* = 0.83, *t*(788) = 21.78, *p* < 0.001, *d* = 1.55). We thereby find support for H7.

Finally, we investigated a potential interaction effect between source similarity and credibility. We ran a 2 × 2 factorial ANOVA on data from study 4, which revealed both a significant main effect of source credibility (*F*(1,786) = 508.25, *p* < 0.001, *η*_*p*_^*2*^ = 0.39), and source similarity (*F*(1,786) = 54.08, *p* < 0.001, *η*_*p*_^*2*^ = 0.06). The interaction between source credibility and similarity was also significant (*F*(1,786) = 15.32, *p* < 0.001, *η*_*p*_^*2*^ = 0.02), with post-hoc comparisons showing that when source credibility was low, source similarity did not have a significant effect on perceived reliability of false headlines (*M*_*diff*_ = − 0.23, *p* = 0.07, *d* = − 0.24), whereas when source credibility was high, source similarity had a significant effect (*M*_*diff*_ = 0.75, *p* < 0.001, *d* = 0.80). Post-hoc comparisons further showed that when the source was similar, credibility had a larger effect (*M*_*diff*_ = 1.76, *p* < 0.001, *d* = 1.88) compared to when the source was dissimilar (*M*_*diff*_ = 1.24, *p* < 0.001, *d* = 1.33), but credibility was significant across both levels of similarity (see Fig. 6).

#### The impact of sources on perceived reliability of factual headlines

Using data from study 3, a one-way ANOVA with source similarity as the IV and perceived reliability of factual headlines as the DV was found significant (*F*(2,7695) = 80.12, *p* < 0.001, *η*_*p*_^*2*^ = 0.02), with post-hoc tests revealing that individuals judged factual headlines as more reliable when they were attributed to a politically similar source compared to dissimilar (*M*_diff_ = 0.44, *p* < 0.001, *d* = 0.33). In study 4, a factorial ANOVA with credibility and similarity as the IVs and perceived reliability of factual headlines as the DV showed no significant effect of similarity (*p* = 0.09), but an effect of credibility (*F*(1,786) = 328.33, *p* < 0.001, *η*_*p*_^*2*^ = 0.29), with perceived reliability being higher for news outlets described as high in credibility compared to low (*M*_diff_ = 1.29, *SE* = 0.07, *p* < 0.001, *d* = 1.29). There was no interaction between credibility and similarity (*p* = 0.31).

Across 5 studies, we thus show that contextual cues *sometimes* influence judgements of misinformation. In study 2 we find evidence that individuals are more likely to believe misinformation when they are exposed to the judgements of another group (either previous participants judgements or via social media comments), leading to an increase in estimations of wider social consensus (perceived consensus), which in turn increases the individuals’ own perceptions of news veracity. However, we find no effect of numbers of likes, retweets and comments on neither perceived consensus, nor individual misinformation susceptibility. Furthermore, in studies 3 and 4 we find that similarity with news sources, whether real or fictitious, impacts the perceived reliability of misinformation, with politically similar sources being viewed as more persuasive, but only if they are credible news outlets. Table 1 shows an overview of the hypotheses tested in studies 1–4.

**Table 1:** Overview of hypotheses and results by study.

Hypothesis (H)	Study & manipulation	DV	Result	Support for H
(1) Social cues impact susceptibility to misinformation	(1a) Likes, retweet & comment numbers	Perceived reliability	*F*(2,7785) = 42.64, *p* < 0.001	✓
(1) Social cues impact susceptibility to misinformation	(1b) Likes, retweet & comment numbers	Perceived reliability	*F*(2,625) = 2.58, *p* = 0.08	✕
(1) Social cues impact susceptibility to misinformation	(2) Explicit (Previous participant judgements) & Implicit (Comments)	Perceived reliability	*F*(4,725) = 6.35, *p* < 0.001	✓
(2) Social cues impact perceptions of social consensus	(1b) Likes, retweet & comment numbers	Perceived social media consensus	*F*(2,624) = 1.01, *p* = 0.37	✕
(2) Social cues impact perceptions of social consensus	(2) Explicit (Previous participant judgements) & Implicit (Comments)	Perceived public consensus	*F*(4,725) = 51.84, *p* < 0.001	✓
(3) Perceived consensus predicts perceived reliability of misinformation	(1b) Likes, retweet & comment numbers	Perceived social media consensus	Adjusted *R*^2^ = 0.19, *F*(1,625) = 143.8, *p* < 0.001	✓
(3) Perceived consensus predicts perceived reliability of misinformation	(2) Explicit (Previous participant judgements) & Implicit (Comments)	Perceived public consensus	Adjusted *R*^2^ = 0.14, *F*(1,728) = 118.2, *p* < 0.0001	✓
(4) Perceived consensus mediates the effect of social cues on perceived reliability of misinformation	(2) Explicit (Previous participant judgements)	Perceived public consensus	Indirect effect: *b* = 0.63, *p* < .001, 95% CI [0.43, 0.86]	✓
(4) Perceived consensus mediates the effect of social cues on perceived reliability of misinformation	(2) Implicit (Comments)	Perceived public consensus	Indirect effect: *b* = 0.21, *p* < 0.001, 95% CI [0.12, 0.34]	✓
(5) Sources influence perceived reliability of misinformation	(3) News headlines with vs without sources	Perceived reliability	*t*(10,539) = − 6.71, *p* < 0.001, *d* = − 0.15)	✓
(6) Source similarity increases perceived reliability of misinformation	(3) Real news sources (politically similar vs dissimilar vs control)	Perceived reliability	*F*(2,7695) = 31.31, *p* < 0.001	✓
(6) Source similarity increases perceived reliability of misinformation	(4) Fictitious sources (similar vs dissimilar)	Perceived reliability	*t*(788) = − 6.18, *p* < 0.001, *d* = − 0.44	✓
(7) Source credibility increases perceived reliability of misinformation	(4) Fictitious sources (high credibility vs low credibility)	Perceived reliability	*t*(788) = 21.78, *p* < 0.001, *d* = 1.55	✓

## Discussion

This work provides novel insight into the link between individual cognition and social context, and highlights how socio-cognitive factors can increase individual susceptibility to misinformation. Combining the results from five studies, we here outline the contextual factors that affect judgements of misinformation, and most importantly the mechanisms through this influence occurs.

Our results provide evidence that both social cues and source cues influence misinformation susceptibility, but only under certain conditions. Results show that explicit cues indicating the judgements of others had a particularly pronounced effect on misinformation susceptibility. Implicit cues endorsing the reliability of the headline also influenced misinformation susceptibility, but only when excluding participants who failed the attention check. It may be that those who failed the attention check were not sufficiently paying attention to the social cue information—in particular in the implicit conditions that required reading several comments. This discrepancy, however, further cements the point that social cues may *only* influence misinformation susceptibility if these cues are attended to and influence wider social consensus perceptions.

Although the correlational mediation results are suggestive, but not conclusive, the results give rise to the possibility that social cues may be more likely to influence misinformation susceptibility if the cues impact the individuals’ perceptions of a wider social consensus. That is, our work suggests that being exposed to judgements of “others” increases individual misinformation susceptibility *only* if those others judge misinformation to be reliable and if this exposure impacts perceived social consensus. The weight news consumers assign to the social cues they are exposed to in relation to whether these are seen as representative of a wider social consensus may differ not just based on the cues themselves, but also based on the individual news consumer.

Through the first highly powered large-scale experiment with a control condition, this work also provides a potential explanation for why engagement cues such as ‘likes’ may *not* impact susceptibility: Because they may not influence perceptions of wider social consensus. We find that perceived consensus not only predicted susceptibility to misinformation, but also mediated the relationship between social cues and individual judgements. That is, being exposed to social cues that indicate the judgements of others led to increased wider social consensus perceptions, which in turn predicted individual susceptibility. Although the mediation model does not offer causal evidence for the relationship between perceived consensus and misinformation susceptibility (e.g. see Coenen^52^), it does offer a potential explanation for the underlying persuasive mechanism of social cues. Being exposed to social cues from a limited group (e.g., previous participants in a study) might lead individuals to infer judgements of a much larger group (e.g. the general public). These judgements may in turn influence the individuals’ own judgements. However, an alternative explanation could be that people who perceive misinformation to be reliable in the first place may, in a form of false consensus effect, also perceive a greater level of public consensus. Future research could seek to disentangle these two explanations.

Taken together, our findings suggest that when social media users are exposed to social cues that indicate that others believe misinformation, this leaves them more vulnerable to misjudgeing the information as reliable. While following the majority can be a reliable heuristic, as statistically speaking the majority is often more likely to be correct^53^, it may be an unreliable cognitive bias when individuals extrapolate information from a small selection of news consumers they are exposed to on social media. Given the existence of echo-chambers^54^, where users mainly interact with and are exposed to information from likeminded others, this could undermine the wisdom of crowds effect by reducing the diversity of viewpoints and creating “misinformation bubbles”, leaving some social media users more predisposed to being influenced by misinformation. This further suggests that individuals can contribute to the influence of misinformation on other news consumers, as their visible judgements may in turn influence the judgements of others.

Surprisingly, results showed that both high and low numbers of ‘likes’ underneath headlines were associated with reduced misinformation susceptibility compared to the control condition. We speculate that this may be caused by these cues making the news items appear more ‘social media like’, leading to a greater level of distrust in the content. For example, research has shown that people who value news literacy are more skeptical of general information quality on social media^55^. Given that study 1a recruited participants via a platform on which people can learn about misinformation, this could explain the observed effect. This assessment is further supported by the finding that even true news headlines were judged as less reliable in both the high and low ‘likes’ conditions compared to the control; however future research is needed to further unpack this.

Interestingly, when comparing the results for misinformation judgements to those of factual headlines, results showed that here, only discrediting reduced perceived reliability in relation to the control condition—and only for *implicit* cues, rather than *explicit* cues. This suggests that individuals openly questioning facts on the internet may lead other news consumers to question valid news and reporting, whereas social cues discrediting *mis*information did not seem to sway judgements. There could be several explanations for this result, but we suggest the possibility that social cues perhaps only impact beliefs when they contrast with personal judgements. That is, social proof, or the influence of others, may be particularly potent when it challenges preexisting beliefs, or when there is a discrepancy between one's own judgement and the perceived judgement of a social group. In other words, news consumers may already have a good sense of what is true and false—until they are exposed to social cues that challenge their own assessments.

Our results also echo previous research that has found that group identity of sources impacts individual susceptibility to misinformation^26^. We show that people’s political or ideological congruence with both real and fictitious news sources increases misinformation susceptibility. The effect with fictitious sources shows that judgements are not purely driven by previous experiences with familiar sources. That is, the rejection of misinformation from dissimilar sources and the acceptance of misinformation from similar sources cannot simply be attributed to previous exposure and knowledge of specific sources that one deems unreliable.

Interestingly, results also clearly show that despite political source similarity playing a significant role in perceived reliability of misinformation, the vignette and credibility metre descriptions of the source were substantially more important. Indeed, we find that similar sources did not have a differentially larger effect than dissimilar sources on misinformation susceptibility when source credibility was low. This indicates that if news consumers know that a news source lacks credibility and journalistic principles, they do not fall for misinformation from that source *simply* because it is politically similar.

The finding that news consumers are less critical of information from sources they believe are credible can in some respects be seen as a positive finding, as it suggests that individuals prioritise adherence to principles of truthfulness and accuracy. Lay people and crowds may, on average, be good at assessing the credibility of news outlets^56,57^. However, this ‘wisdom of crowds’ effect can be undermined by the fact that crowds tend to challenge only the credibility of the those who they politically disagree with^58^. Indeed, as previous research has found that credibility judgements can be subjective^26^, this can also be problematic, for instance, if one deems sources such as Breitbart—a source that researchers have identified as one playing a significant role in misinformation spread^50^—as credible. Unfortunately, otherwise reputable mainstream sources *sometimes* publish misinformation^59^, which this work suggests could be much more influential than extreme falsehoods from illegitimate news sources, given people’s tendency to use source cues when forming judgments about misinformation. As misinformation producers use a variety of manipulation techniques, including misleading credibility cues, this presents an ongoing issue for modern news consumption.

### Limitations

Although we assessed the overall question of whether and how social context influences misinformation susceptibility across five highly powered studies, future research may extend this work in a few different ways. Firstly, in order to assess causality in the direction of the relationship between social cues and misinformation susceptibility, this study was conducted using simulated social media environments and not on real social media platforms where social processes are undoubtedly more complex. The social context of information consumption is multifaceted in itself, and there are many unexplored social psychological variables that may also be at play. For instance, it may be the case that group identity processes interact with social consensus cues, making ingroup majorities more persuasive than outgroup majorities. Future work could seek to explore potential compounding effects. Indeed, although we find that perceived social consensus mediated the relation between social cues and misinformation susceptibility, there may be other mediators that play a role in this process that we did not explore.

Although we measured sharing intentions in some of the studies (see Supplementary Information Table G), we focused on perceived reliability of misinformation to study misinformation susceptibility. However, the dangers of misinformation do not stop at reliability judgements. It is possible that repeated exposure to misinformation that is judged as unreliable still influences beliefs (e.g., about science, politics, or health) or behaviour. Finally, although our samples were balanced on various demographic variables, they were not representative of any country’s population. A requirement for participation was being English-speaking, but as English may not have been the first language of all participants, that several of the social cues (in particular, the comments in study 2) were written in English may have impacted their influence effects.

Despite these limitations, this research contributes to the debate on the influence of credibility cues^27^ and social biases. The results provide clear evidence social context plays a significant role when individuals make judgements about the reliability of misinformation. This paper highlights that it is not sufficient to study cognitive processes involved in news consumption completely removed from the socio-cognitive context in which they occur.

## Methods

All additional measures and scales collected as part of each study can be found in the Supplementary Information (Table H). Here we present the measures included in the paper analyses. All studies and experimental protocols were approved by the Cambridge Psychology Research Ethics Committee. All methods were performed in accordance with the relevant guidelines and regulations.

### Study 1a

#### Purpose

The purpose of study 1a was to explore whether engagement cues on social media platforms influence perceived reliability of misinformation.

#### Participants

In study 1a, data was collected as part of a larger intervention experiment on social cues using the *Bad News* platform^60^, and data for study 1a consisted of data from the pre-intervention questions. This study relied on press coverage for *Bad News* (https://www.getbadnews.com/) which allowed anyone with access to the internet to visit the website and participate in the study. As such, this study used a convenience sample. Data collection was set to run for as long as the game platform was available to host the study—between 21st December 2018 and 11th April 2019. The final sample was *N* = 7788, 58% between 18 and 29, 39% female, 85% completed at least some college, 48% left-leaning, 90% used social media regularly or daily. The study was approved by the Cambridge Psychology Research Ethics Committee (PRE.2018.085).

#### Design and materials

##### Social cue manipulation

In the high condition (*n* = 1349), likes and retweet numbers were set to be > 1000 and comments > 100, whereas in the low condition (*n* = 4411) all values were set < 10. In the control condition (*n* = 2028) these measures were cropped out.

##### Headlines

In study 1a we created fictitious false headlines based on 6 misleading strategies used by misinformation producers based on previous research^60^. The creation of headlines allowed us to ensure participants had not seen the headlines before, and secondly, to embed the misinformation techniques in a highly controlled manner. These six misinformation tactics included (1) using *impersonation tactics* to mislead individuals to think the source of the information is credible, (2) *polarising* audiences by harnessing the divide between political groups and purposefully attacking outgroups, (3) using exaggeratedly *emotional* language to distort the news story to generate strong emotional responses, (4) creating or inspiring *conspiratorial thinking* to rationalise current events, (5) *trolling* users, famous people or organisations to either disrepute them or erroneously link them to recent news, and (6) *discrediting* otherwise reputable individuals, institutions or facts to instill doubt in audiences.

Two ‘reset’ headlines were included in each condition, in which social cues represented the opposite of the assigned condition, to ensure that consensus tweets would be relatively high in comparison to the social cues in the assigned condition. For analyses, we only included the headlines that matched the participants’ condition. Participants were also shown 2 factual control headlines, which did not employ any deceptive techniques. Control headlines did not employ any misinformation strategies and the content of these was chosen to reflect global news events. An overview of the items used can be found in the Supplementary Information (Table A).

#### Measures

##### Perceived reliability of misinformation

The main dependent variable was participants ability to recognise misinformation in the form of misleading headlines. Participants were asked to rate the reliability of headlines on a 7-point Likert scale, where 1 = very unreliable and 7 = very reliable. For an extensive discussion of the use of different question framings (e.g., how manipulative/ reliable/ accurate/ trustworthy do you find this headline) and response modes (e.g., binary, 6-point or 7-point scales), see Roozenbeek et al.^61^. Participants were presented with 6 deceptive headlines with social cues in line with their condition (e.g. if in the high condition, participants saw 6 deceptive headlines with high numbers, 1 deceptive ‘reset’ with low numbers, 1 control ‘reset’ with low numbers, and 1 control with high numbers.

##### Demographics

Age was measured categorically (18–29, 30–49 and over 50). Highest level of completed education was categorised as “high school or less”, “college or university”, “higher degree” and gender as “female”, “male” and “other”. Political affiliation was measured on a 7-point scale where 1 = very left-wing and 7 = very right-wing. Finally, data on current use of *Twitter* was collected (“I don’t have an account”, “I have an account, but I hardly ever use it”, “I have an account, and I use it occasionally”, “I have an account and I use it often”, “I have an account and I use it on a daily basis”).

#### Procedure

Participants who visited the website for the *Bad News*^60^ game between 21st December 2018 and 11th April 2019 were asked if they would like to take part in a scientific study prior to gameplay. This game is a psychological intervention that aims to train participant to resist falling for misinformation. When individuals decide to play the game, the platform allows researchers to recruit participants to a scientific research study prior to gameplay. The platform is set up to simulate a social media feed presenting participants with what appears to be a Twitter-like timeline, where various news headlines or “posts” appear. The platform has been used in a variety of misinformation studies as a data collection platform.

If participants agreed to take part in our study, they were asked to provide informed consent. Following this, they answered the socio-demographic questions. As automatic randomisation to conditions was not possible on the platform, participants were assigned to a condition by selecting a number from 1 to 3, which then redirected them to one of the three social cue conditions. Once they were assigned a condition, they were exposed to news headlines and told that these were screenshots of real headlines, and asked to report on the above measure (perceived reliability). After completing the study, participants were debriefed regarding the true purpose of the study and informed that the headlines were fictitious.

### Study 1b

#### Purpose

The purpose of study 1b was to explore the unanswered questions in study 1a: Firstly, whether social cues influence perceptions of social consensus, and secondly whether social cues influence perceived reliability of misinformation when controlling for potential source effects.

#### Participants

A power analysis indicated a minimum sample size of 609 participants was necessary to capture small effects (*d* = 0.30) with 95% power at an alpha level of 0.05, and as such we recruited *N* = 628 participants via *Prolific* (68% females, *M*_age_ = 37, 50% university educated, 52% left-leaning, 74% had a *Twitter* (now *X*) account). Participants from anywhere in the world could participate in the study, but a requirement for participation was being fluent in English. Participants were randomly assigned to conditions: High (*n* = 212), low (*n* = 206), or the control (*n* = 210). All participants provided informed consent and were compensated for their time. The study was approved by the Cambridge Psychology Research Ethics Committee (PRE.2018.085). All methods were performed in accordance with the relevant guidelines and regulations.

#### Design and materials

##### Social cue manipulation

In the high condition, likes and retweet numbers were set to be > 1000 and comments > 100, whereas in the low condition all values were set < 10. In the control condition these measures were cropped out.

##### Headlines

To identify “real” misinformation that had previously been posted online, we relied on the 6 misinformation strategies used in study 1a. To select unreliable news headlines, the platform known as “Hoaxy” was used: A platform developed by researchers at Indiana University, which visualises the spread of claims and fact checking. For the categories “*Impersonation*” and “*Trolling*”, the appropriate headline could not be found through the Hoaxy platform given the nature of these misinformation techniques, and a manual *Twitter* search was carried out instead. Two control questions were included, which did not make use of deceptive techniques or strategies, and the content of which was chosen to reflect factual global news events among English speakers.

To control for potential source effects the source was blurred out, apart from the headlines employing the impersonation strategy, as the basis of impersonation lies in impersonating a real person, which would not be feasible to represent without a source.

#### Measures

##### Perceived reliability of misinformation

To measure misinformation susceptibility, participants were asked to rate each headline’s reliability on a standard 7-point Likert scale: *“On a scale from 1 to 7, how reliable do you find this tweet”* (1 = very unreliable, 7 = very reliable).

##### Intention to share

Participants were asked to indicate how likely they would be to share each item on social media on a 7-point Likert scale: *“On a scale from 1 to 7, how likely is it that you would share this with your network, if it came up on your newsfeed?”* (1 = very unlikely, 7 = very likely).

##### Perceived consensus

Participants were asked to indicate what percentage of other *Twitter* users they believed would find the item reliable: *“What percentage of Twitter users do you think would find this tweet reliable?”* (0–100% of *Twitter *users).

##### Demographics

Socio-demographic variables included gender (male, female, other), age (birth year), political orientation (measured on a 7-point Likert scale where 1 is very left-wing and 7 is very right-wing), highest level of education completed (less than high school degree, high school graduate, bachelor’s degree, master’s degree, doctoral degree or professional degree), current use of *Twitter *(“I don’t have an account”, “I have an account, but I hardly ever use it”, “I have an account, and I use it occasionally”, “I have an account and I use it often”, “I have an account and I use it on a daily basis”) and news consumption source (“I don’t really follow the news”, “social media”, “TV and radio”, “print media” (newspapers, magazines), “word of mouth”).

#### Procedure

Participants were recruited via Prolific, where the study was advertised as a study on “News Perception”. Requirements for participating was that they had not previously participated in studies run by the same lab, that they were above 18 years old and that they were fluent English-speakers. Participants from anywhere in the world could take part.

Once they were redirected to the *Qualtrics* platform, they were shown information about the study and provided informed consent prior to starting. Then, participants answered the demographic questions. Following this, participants were randomly assigned to one of the three conditions. They then proceeded to answer the questions for each headline, which were presented in random order. The only two headlines, for which the order was set, were polarisation headlines, as these were presented first and last, counterbalanced for political orientation. Following the answering of the main questions, participants proceeded to complete two additional psychological scales (for a full overview of measures included in each study, see Supplementary Information Table H). Finally, participants were debriefed.

### Study 2

#### Purpose

The purpose of study 2 was to assess firstly whether explicit and implicit social cues influence perceived reliability of misinformation and secondly, whether these cues influence perceived consensus in misinformation reliability.

#### Participants

Participants were recruited on *Prolific* and again invited to participate in a study on “News Perception”. A requirement for participating was being fluent in English, but other wise participants from all over the world could parttake. For this study, a power analysis indicated *N* = 470 participants would be sufficient to detect a small effect size of *d* = 0.40 with five groups, an alpha level of 0.05 and 95% power. The final sample was *N* = 730 participants (46% female, *M*_*age*_ = 27, 53% university educated, 64% left-leaning, 94% had a *Facebook* account, 46% got majority of news from social media and 32% from online news sites) remaining across the five conditions: Control condition (*n* = 148), implicit discrediting (*n* = 146), implicit endorsement (*n* = 146), explicit discrediting (*n* = 145) and explicit endorsement (*n* = 145). As specified in the pre-registration, we analysed results with the full sample as well as with only those who passed an attention check. All participants provided informed consent and were compensated for their time. The study was approved by the Cambridge Psychology Research Ethics Committee (PRE.2020.059). All methods were performed in accordance with the relevant guidelines and regulations.

#### Design and materials

##### Social cue manipulation

We manipulated social cues across the following 5 conditions. We firstly manipulated social cue format: *explicit* vs *implicit* and direction: *endorsement* vs *discrediting*. We also included a control condition with no social cues.

To manipulate *explicit* consensus, participants were explicitly informed about the judgements of an unidentified previous group of ‘other’ participants. This involved showing participants a statement before exposure to each headline that read: “X percent of previous participants judged the following headline to be reliable (*endorsement* condition)/unreliable (*discrediting* condition)”. This percentage was always above 72%.

To manipulate *implicit* consensus, participants were exposed to five comments underneath each headline which all either implied the individual commenter believed (*endorsement* condition), or did not believe (*discrediting* condition) the headlines.

##### Headlines

Participants were exposed to three misinformation headlines and one control (factual) headline. The misinformation used relied on deception techniques rather than being blatant “fake news”. Here, we again identified misinformation items using the misinformation platform Hoaxy. We included items that had been published in the last year and made use of the emotional language manipulation technique. This time, we used a *Facebook* layout as opposed to the *Twitter* layout used previously. An overview of all items used in this study can be found in Supplementary Information Table C.

##### Demographics

Socio-demographic variables included gender (male, female, other), age (birth year), political orientation (measured on a 7-point Likert scale where 1 is very left-wing and 7 is very right-wing), highest level of education completed (less than high school degree, high school graduate, bachelor’s degree, master’s degree, doctoral degree or professional degree), current use of *Facebook* (“I don’t have an account”, “I have an account, but I hardly ever use it”, “I have an account, and I use it occasionally”, “I have an account and I use it often”, “I have an account and I use it on a daily basis”) and news consumption source (“I don’t really follow the news”, “social media”, “TV and radio”, “print media” (newspapers, magazines), “word of mouth”, and “online news sites (excluding social media)”.

#### Measures

This study investigated additional measures than those listed here. Please see Supplementary Information (Table H) for additional measures in this study.

##### Perceived reliability of misinformation

To measure misinformation susceptibility, participants were asked to rate each item’s reliability on a 7-point Likert scale: *“On a scale from 1 to 7, how reliable do you find this tweet”* (1 = very unreliable, 7 = very reliable).

##### Perceived consensus

Participants were asked to indicate what percentage of the general population they believed would find the headline reliable: *“What percentage of the general population do you think would find this headline reliable?”* (0–100%).

##### Intention to share

Participants were asked to indicate how likely they would be to share each item on social media on a 7-point Likert scale: *“On a scale from 1 to 7, how likely is it that you would share this with your network, if it came up on your news feed?”* (1 = very unlikely, 7 = very likely).

##### Attention check

At the end of the study, all participants were asked to state whether the social cues they were exposed to indicated that other individuals found the information reliable or unreliable: “*Did the information you saw about the responses of other individuals indicate that the majority judged the headlines to be reliable or unreliable?”* with response options being “Reliable”, “Unreliable”, or “I didn’t see any such information”.

#### Procedure

Participants were recruited via *Prolific*, where the study was advertised as a study on “News Perception”. Requirements for participating was that they had not previously participated in studies run by the same lab, that they were above 18 years old and that they were fluent English-speakers.

Once participants were redirected to the *Qualtrics* platform, where the study was hosted, they were provided with information about the study and provided informed consent prior to starting. Then, participants answered the demographic questions. Following this, participants were randomly assigned to one of the five conditions. They then proceeded to answer the questions for each headline, which were presented in random order. Finally, participants were debriefed.

### Study 3

#### Purpose

The purpose of study 3 was to investigate whether source cues impact misinformation susceptibility using a large sample size and a between-subject design. The study was approved by the Cambridge Psychology Research Ethics Committee (PRE.2019.104). All methods were performed in accordance with the relevant guidelines and regulations.

#### Participants

As in study 1a, data for study 3 was collected on the *Bad News* platform^60^ as part of a larger intervention experiment on source effects, and data for study 3 consisted of data from the pre-intervention questions. This study again relied on press coverage for the *Bad News* game, which allowed anyone with access to the internet to visit the website and participate in the study. As such, this study used a convenience sample. Data collection was set to run for as long as the game platform was available to host the study—between 1st November 2020 and 15th January 2021. The final sample was *N* = 10,541 (60% between 18 and 29, 44% female, 49% higher educated, 52% liberal).

#### Design and materials

This study employed a between-subjects design and manipulated source slant across three conditions (liberal (*n* = 6184) conservative (*n* = 2608) and control (*n* = 1749)).

##### Source manipulation

We manipulated the news source on *Twitter *news headlines across three conditions: A traditionally liberally slanted news source (CNN, the Washington Post or the New York Times), a conservatively slanted news source (Wall Street Journal, Fox News and Breitbart) or a control condition where the source was blurred out.

##### Headlines

The false headlines employed one of three manipulation techniques of ‘conspiratorial thinking’, ‘discrediting opponents’ and ‘emotional language’ previously discussed. The factual headlines did not make use of any manipulation techniques and were based on factual news. An overview of all headlines in this study can be found in Supplementary Information Table D.

#### Measures

This study investigated additional measures than those listed here. Please see Supplementary Information (Table H) for additional measures in this study.

##### Perceived reliability of misinformation

To measure misinformation susceptibility, participants were asked to rate each item’s reliability on a standard 7-point Likert scale: *“How reliable is the above news headline”* (1 = very unreliable, 7 = very reliable).

##### Source similarity

Source similarity was coded according to whether or not the participants’ reported political ideology matched the political slant of the source. As political affiliation was originally collected on a 7-point scale, this was converted to a binary (liberal vs conservative) measure. For the analyses which focused specifically on source similarity, participants who had reported a ‘moderate’ political stance (that is, a 4 on the 1–7 scale) were excluded.

##### Demographics

Age was measured categorically (18–29, 30–49, and over 50). Highest level of completed education was categorised as “high school or less”, “college or university”, “higher degree” and gender as “female”, “male” and “other”. Finally, political affiliation was measured on a 7-point Likert scale where 1 = very left-wing and 7 = very right-wing.

#### Procedure

Participants who visited the website for the *Bad News*^60^ game between 1st November 2020 and 15th January 2021 were automatically asked if they would like to take part in a scientific study prior to gameplay. If participants agreed to take part in our study, they were asked to provide informed consent. Following this, they answered the socio-demographic information. As automatic randomisation to conditions was not possible on the game platform, participants were assigned to a condition by selecting a number from 1 to 3, which redirected them to one of the three source conditions. Once they were assigned a condition, they were exposed to new headlines and told that these were screenshots of real headlines, and asked to report on the above measures (perceived reliability). After completing the study, participants were debriefed regarding the true purpose of the study and informed that the headlines were fictitious.

### Study 4

#### Purpose

The purpose of study 4 was to examine the direct influence of source credibility and similarity on misinformation susceptibility.

#### Participants

*N* = 790 US participants were recruited on *Prolific* to participate in a study on “News Perception” (65% female, *M*_*age*_ = 35, 62% completed higher education, 52% liberal, 86% had a Facebook account, 34% got majority of their news from social media). All participants provided informed consent and were compensated for their time. The study was approved by the Cambridge Psychology Research Ethics Committee (PRE.2021.066). All methods were performed in accordance with the relevant guidelines and regulations.

#### Design and materials

This study employed a 2 × 2 between-subjects factorial design manipulating source similarity (similar vs dissimilar) and source credibility (high credibility vs low credibility).

##### Source similarity

We manipulated source similarity by providing descriptions of the source which either described the source as having a primarily liberal or conservative slant. This description was presented alongside a metre, that visualised the slant of the source on a ‘Liberal’ to ‘Conservative’ spectrum. We then coded the source in line with the participants’ reported political ideology, such that the source was either similar (e.g. liberal source and liberal participant) or dissimilar (e.g. conservative source and liberal participant).

##### Source credibility

Source credibility was manipulated by describing the source as either as a) being known to publish news stories that adhere to journalistic principles of truthfulness and accuracy or b) being known to publish questionable news stories that have little basis in fact. This was also visualised in a metre ranging from 'Credible’ to ‘Not Credible’.

##### Headlines

Participants were exposed to 5 misinformation headlines and 5 factual headlines. These headlines were similar to those used in study 4 and made use of one of three misleading tactics of invoking conspiratorial thinking, discrediting and using overly emotional exaggeration. The 5 factual headlines included two headlines which had previously been used in study 4, but this time we also included three new factual headlines, which again were based on current factual events and which did not make use of any misleading elements. An overview of all headlines used in this study can be found in Supplementary Information Table E.

#### Measures

##### Perceived reliability of misinformation

As in the previous studies, we collected data on the perceived reliability of headlines by asking participants to report: *“How reliable is the above news headline”* (1 = very unreliable, 7 = very reliable).

##### Demographics

Participants answered demographics questions including age (year of birth), education (less than high school, high school graduate, bachelor’s degree, master’s degree, doctoral degree, professional degree), sex (male, female, other), political affiliation (7-point Likert scale arranged from extremely liberal (left) to extremely conservative (right)), political party preference (strongly democratic, democratic, lean democratic, lean republican, republican, strongly republican), *Facebook* use (“I don’t have an account”, “I have an account, but I hardly ever use it”, “I have an account, and I use it occasionally”, “I have an account and I use it often”, “I have an account and I use it on a daily basis”) and news consumption source (“I don’t really follow the news”, “social media”, “TV and radio”, “print media” (newspapers, magazines), “word of mouth”, and “online news sites (excluding social media)”.

##### Attention check

At the end of the study we included an attention check to ensure participants had read the source descriptions. Participants were asked two questions with the first being: *“How was the credibility of News Publisher A described?”* with response options being: (1) “News Publisher A was described as a mainstream media source, which is generally known to publish news stories that adhere strictly to journalistic principles of truthfulness and accuracy”, (2) “News Publisher A was described as a media source, which is generally known to publish questionable news stories that have little basis in fact” or (3) “I didn't see any such information”. The second attention check question was: *“How was the political slant of News Publisher A described?”*with response options being: (1) “News Publisher A was described as primarily having a liberal editorial slant”, (2) “News Publisher A was described as primarily having a conservative editorial slant” or (3) “I didn't see any such information”.

#### Procedure

Participants were recruited via *Prolific*, where the study was advertised as a study on “News Evaluation”. Requirements for participating was that they had not previously participated in studies run by the same lab, that they were above 18 years old, that they were fluent English-speakers, and that they identified as either Conservative or Liberal.

Once they were redirected to the *Qualtrics* platform, where the study was hosted, they were provided with information about the study and provided informed consent prior to starting. Then, participants answered the demographic questions. Following this, participants were randomly assigned to one of the four conditions.

Before being asked to rate the reliability of headlines, participants were shown the source vignettes and metres in line with their condition. They then were presented with the 10 news headlines in randomised order and asked to rate the reliability of each of the headlines. Following the main experiment, participants proceeded to complete psychological scales (for an overview of all scales used see Table H in Supplementary Information) and then answered the demographic questions. At the end, they completed the attention check. Finally, participants were debriefed.

### Supplementary Information

Supplementary Tables.

## Data Availability

The datasets analysed during the current study are available in the OSF repository: https://osf.io/pfa7w/?view_only=8cc58a2bc5744c84b10033fcde699900.
