# Supplementary material for: The persuasive effects of social cues and source effects on misinformation susceptibility
Source: Sci Rep. 2024 Feb 20;14:4205. doi: 10.1038/s41598-024-54030-y (PMC10879158; doi:10.1038/s41598-024-54030-y)
Supplement: Supplementary file 1 — Supplementary Tables. [file 41598_2024_54030_MOESM1_ESM.docx]

**Supplementary Information**

**Table A: Full overview of items used in Study 1a**

| **Misinformation vs Facts** | **Category** | **Content** | **Source Name** |
| --- | --- | --- | --- |
| Misinformation | Conspiracy | The Bitcoin exchange rate is being manipulated by a small group of rich bankers. #InvestigateNow | The Daily Web News @dailyweb |
| Misinformation | Emotion | News alert: baby formula caused HORRIFIC outbreak of new terrifying disease among helpless infants. Parents despair. #BabyScandal | Parents Weekly @ParentsWeekly |
| Misinformation | Discrediting | The mainstream media has been caught in so many lies that it can’t be trusted as a reliable news source. #FakeNews | Rapid Updates @RapidUpdates |
| Misinformation | Polarisation | The myth of “equal IQ” between left-wing and right-wing people exposed. #TruthMatters | Asco @Asco |
| Misinformation | Trolling | Hey @LeoDicaprio. It’s snowing and freezing in New York. Could use some of that global warming you’re always going on about! #IceCold | Capunia & Co. @CapuniaCo |
| Misinformation | Impersonation | The 8^th^ season of Game of Thrones will be postponed due to a salary dispute. #GameOfThrones | HBÖ @hbö |
| Misinformation | Discredit (reset) | Medical students only receive a total of 5 hours of tutoring in nutrition. Don’t trust doctors’ dietary advice. | Susan P @SusanP |
| Factual Information | - | Donald Trump wants to build a wall between the US and Mexico. | The New York Times @nytimes |
| Factual Information | (reset) | Apple, Google and Amazon named as most valuable brands in the world. | The Guardian @guardian |

**Table B: Full overview of items used in Study 1b**

| **Misinformation vs Facts** | **Category** | **Content** | **Source Name** |
| --- | --- | --- | --- |
| Misinformation | Conspiracy | Uber Creepy: Tracking Your Every Move | Hidden |
| Misinformation | Conspiracy | Exposing the shadow “elite” controlling the world. | Hidden |
| Misinformation | Emotion | Horrific’ TV show inspiring suicide, says filmmaker | Hidden |
| Misinformation | Emotion | Cop arrested after bragging about video of unprovoked attack on helpless man | Hidden |
| Misinformation | Discredit | No Scientific Basis for Airport Behavior Screening – TSA Internal files | Hidden |
| Misinformation | Discredit | Scandal: MRI Brain-imaging completely unreliable | Hidden |
| Misinformation | Polarisation | Professor Calls ‘Left(/Right)-Wing’ Reporting a Form of ‘Violence | Hidden |
| Misinformation | Polarisation | 92% of left(/right)-wing activists live with their parents, Study: 1 in 3 also unemployed | Hidden |
| Misinformation | Trolling | @algore: Hey bro! When can we expect to get some of this global warming= It’s getting kinda chilly and the snow isn’t much fun. @LeoDiCaprio: Feel free to add you insights as well. | Hidden |
| Misinformation | Trolling | I just learned that @amazon allows anti-vaxxers to designate an anti-vax “charity” as the recipient of their AmazonSmile donations. Killing children for profits! #antivax #amoral #wtf #capitalism | Hidden |
| Misinformation | Impersonation | Am offering free leavers hoodies to MPs who vote with me #Brexit | @MrsTheresaMay |
| Misinformation | Impersonation | “Just for today I’ve decided to give away 3000 ETH to my fans. Simply send 0.5-4.0 ETH to my address and I’ll send back 5-40 ETH.” | @ElonMuskik |
| Factual Information | - | Apple, Google and Amazon named as most valuable brands in the world. | Hidden |
| Factual Information | - | Angela Merkel to step down as German chancellor in 2021. | Hidden |
| Factual Information | - | Physical fitness keeps your brain in good shape: https://bit.ly/2MgTDu | Hidden |

**Table C: Full overview of items used in Study 2**

| **Misinformation vs Facts** | **Category** | **Content** | **Source Name** |
| --- | --- | --- | --- |
| Misinformation | Emotion | Top headline: “Baby formula linked to horrific disease outbreak.” Bottom headline: “Baby formula linked to horrific outbreak of new, terrifying disease among helpless infants” | Hidden |
| Misinformation | Emotion | Top headline: “Make sure these aren’t in your cupboard!” Bottom headline: “Shocking finding: Common vitamin supplements are strongly linked to cancer” | Hidden |
| Misinformation | Emotion | Top headline: “If you own one, return it IMMEDIATELY. Your safety is at risk.” Bottom headline: “WARNING: smartphone (x) causes explosion, leaving elderly pensioner to fight for her life.” | Hidden |
| Factual Information | - | Top headline: “Biden returns the US to the Paris Agreement” Bottom headline: “The US officially rejoins the Paris climate agreement.” | Hidden |

**Table D: Full overview of items used in Study 3**

| **Misinformation vs Facts** | **Category** | **Content** | **Source Name** |
| --- | --- | --- | --- |
| Misinformation | Conspiracy | Uber Creepy: Tracking your every move | Fox News/CNN/ Control |
| Misinformation | Conspiracy | Exposing the shadow “elite” controlling the world | Wall Street Journal/The Washington Post/Control |
| Misinformation | Emotion | Horrific TV show inspiring suicide, says filmmaker | Breitbart News/ New York Times/Control |
| Misinformation | Emotion | Cop arrested after bragging about video of unprovoked attack on helpless man | Fox News/CNN/Control |
| Misinformation | Discredit | Expert: Scientific studies no longer trustworthy | Wall Street Journal/The Washington Post/ Control |
| Misinformation | Discredit | Scandal: MRI Brain imaging completely unreliable | Breitbart News/ New York Times/Control |
| Factual information | - | Angela Merkel to step down as German chancellor in 2021 | CNN/Fox News/Control |
| Factual information | - | Apple, Google and Amazon named as most valuable brands in the world | The Washington Post/Wall Street Journal/Control |
| Factual information | - | Physical fitness keeps your brain in good shape | New York Times/Breitbart News/Control |
| Factual information | - | Olympics: Tokyo finishes building stadium for 2020 | CNN/Fox News/Control |
| Factual information | - | The new Brexit deadline will be January 31 | The Washington Post/Wall Street Journal/Control |
| Factual information | - | Grammy 2020 Awards: Taylor Swift, Lizzo and Beyonce nominated | New York Times/Breitbart News/Control |

**Table E: Full overview of items used in Study 4**

| **Misinformation vs Facts** | **Category** | **Content** |
| --- | --- | --- |
| Misinformation | Conspiracy | Uber Creepy: Tracking your every move |
| Misinformation | Conspiracy | Exposing the shadow “elite” controlling the world |
| Misinformation | Emotion | Horrific TV show inspiring suicide, says filmmaker |
| Misinformation | Discredit | Expert: Scientific studies no longer trustworthy |
| Misinformation | Discredit | Scandal: MRI Brain imaging completely unreliable |
| Factual information | - | Physical fitness keeps your brain in good shape |
| Factual information | - | Apple, Google and Amazon named as most valuable brands in the world |
| Factual information | - | Netflix to include mobile games for subscribers |
| Factual information | - | The Mandalorian and The Crown Tie for Most Nods in This Year’s Emmy Nominations |
| Factual information | - | Abba reunite for Voyage, first new album in 40 years |

**Table F: Study 2 analyses with exclusions applied**

| **Hypothesis (H)** | **Manipulation** | **DV** | **Result** |
| --- | --- | --- | --- |
| (1) Social cues impact susceptibility to misinformation | Explicit (previous group judgements) & implicit social cues (comments) | Perceived reliability | *F*(4,537) = 10.25, *p* < 0 .001. Explicit contrast: *p* < 0.001, *d* = 0.66. Implicit contrast: *p* = 0.01, *d* = 0.34). |
| (2) Social cues impact perceptions of public consensus | Explicit (previous group judgements) & implicit social cues (comments) | Perceived public consensus | *F*(4,537) = 56.56, *p* < .001. Explicit contrast: *p* < 0.001, *d* = 2.07. Implicit contrast: *p* < 0.001, *d* = 0.77. |
| (3) Perceived consensus predicts perceived reliability of misinformation | Explicit (previous group judgements) & implicit social cues (comments) | Perceived public consensus | (Adjusted *R*^2^ = 0.14, *F*(1,540) = 89.92, *p* < 0.001), |
| (4) Perceived consensus mediates the effect of social cues on perceived reliability of misinformation | Explicit social cues (previous group judgements) | Perceived public consensus | Indirect effect: *b* = 0.267, *p* < 0.001, 95% CI [0.144,0.429]. |
| (4) Perceived consensus mediates the effect of social cues on perceived reliability of misinformation | Implicit social cues (comments) | Perceived public consensus | Indirect effect: *b* = 0.715, *p* < 0.001, 95% CI [0.468,0.996]. |

**Table G: Analyses with intent to share misinformation variable**

| **Analysis** | **Study & Manipulation** | **DV** | **Result** |
| --- | --- | --- | --- |
| One-way ANOVA assessing the impact of social cues on intent to share misinformation. | (1b) Social engagement cues (likes, retweet & comment numbers) excluding sources | Intention to share | *F*(2, 625) = 3.16, *p* = .043 |
| One-way ANOVA assessing the impact of social cues on intent to share misinformation. | (2) Explicit (previous group judgements) & implicit social cues (comments) | Intention to share | *F*(4,725) = 1.26, *p* =0.29 |

**Table H: Psychological scales and additional measures collected in each study**

| **Study** | **Measure** | **Scale** |
| --- | --- | --- |
| 2 | Need for Cognition | Participants were asked to complete a six item Need for Cognition scale*.* Unfortunately, due to a data collection error, only data for 5 of the 6 items were collected. |
| 2 | Social Desirability | Participants completed the 20 item Marlowe-Crowne Social Desirability scale desirability. To gather more detailed information than that permitted by a “true or false” scale, the social desirability scale was adapted to be a 1-5 Likert scale. |
| 3 | Perceived Truth of News Items | As a measure of misinformation susceptibility (in addition to Perceived Reliability) participants were asked “On a scale from 1 to 7, how likely is it that the above information is true?” |
| 3 | Perceived Public Truth | In addition to the Perceived Public Reliability measure included in the main analyses, participants were also asked: “What percentage of the general population do you think would judge the information in the headline to be true?” |
| 5 | Response Time | For each reliability judgement of news headlines, data on participants response time was collected including First Click, Last Click, Total Number of Page Clicks and Total Time on Page. |
| 5 | Cognitive Reflection | Participants completed the three item Cognitive Reflection Test. |
| 5 | Need for Cognition | Participants were asked to fill out the 6-item Need for Cognition scale. |

**Table I: Correlation analyses in studies 1b-2**

| **Study** | **Variables** | **df** | **t** | **Pearson correlation** | ***p*-value** |
| --- | --- | --- | --- | --- | --- |
| 1b | Social Cue Condition (high vs low consensus) by Perceived Consensus | 415 | 1.12 | *0.05* | *0.27* |
| 2 | Social Cue (Implicit Discrediting vs Endorsement) by Perceived Consensus | 290 | 4.48 | *0.25* | *<0.001* |
| 2 | Social Cue (Explicit Discrediting vs Endorsement) by Perceived Consensus | 288 | 15.45 | *0.67* | *<0.001* |
| 1b | Perceived Consensus (in Social Cue Conditions High vs Low) by Perceived Reliability | 415 | 8.86 | *0.40* | *<0.001* |
| 2 | Perceived Consensus (in Social Cue Conditions Implicit Discrediting vs Endorsement) by Perceived Reliability | 290 | 1.38 | *0.08* | *<0.001* |
| 2 | Perceived Consensus (in Social Cue Conditions Explicit Discrediting vs Endorsement) by Perceived Reliability | 228 | 4.49 | *0.26* | *<0.001* |
